# Preemptive oral analgesia with steroidal and nonsteroidal anti-inflammatory drugs in periodontal surgery: a systematic review

**DOI:** 10.3389/fphar.2024.1385401

**Published:** 2024-07-15

**Authors:** Lívio Portela de Deus Lages, Cristiane de Cássia Bergamaschi, Luciane Cruz Lopes, Eduardo Gomes da Frota, Marcus Tolentino Silva, Thiago Lima Monte, Rogério Heládio Lopes Motta

**Affiliations:** ^1^ PhD Program, Faculdade São Leopoldo Mandic, Campinas, Brazil; ^2^ Pharmaceutical Science Graduate Course, University of Sorocaba, Sorocaba, Brazil; ^3^ Department of Public Health, Faculty of Health Sciences, University of Brasília, Brasília, Brazil; ^4^ Centro Universitário Uninovafapi, Teresina, Piauí, Brazil; ^5^ Division of Pharmacology, Anesthesiology and Therapeutics, Faculdade São Leopoldo Mandic, Campinas, Brazil

**Keywords:** periodontal surgery, preemptive analgesia, systematic review, anti-inflammatory drugs, dentistry

## Abstract

**Introduction:** Periodontal procedures can promote prolonged intense pain, particularly in clinical situations requiring surgical procedures. In this context, preemptive analgesia has also been assessed for its utility in controlling post-operative pain and discomfort in patients undergoing periodontal invasive procedures. This study assessed the efficacy and safety of preemptive oral analgesia with steroidal and non-steroidal anti-inflammatory drugs in periodontal surgeries.

**Methods:** This systematic review performed a search in the following electronic sources: the Cochrane Central Register of Controlled Trials (CENTRAL), MEDLINE (via PubMed), EMBASE (via Ovid), Web of Science, Virtual Health Library and in clinical trials electronic databases for relevant randomized clinical trials (RCTs); published up to July 2023. Primary outcomes assessed were post-operative pain, edema and trismus. A narrative synthesis of the findings was carried out.

**Results:** Six RCTs, involving a total of 250 participants, were included. The studies reviewed had a high risk of bias, particularly due to allocation concealment and blinding of participants and personnel. The RCTs reported only the outcome pain. The preemptive use of dexamethasone 8 mg, etoricoxib 90 mg or 120 mg and ketorolac 20 mg seems to be more effective for controlling post-operative pain than placebo.

**Discussion:** The anti-inflammatory drugs evaluated proved to be effective for controlling post-operative pain. However, given the limitations regarding lack of studies, methodological biases, disparities in drugs and doses, report restricted the pain outcome; further RCTs confirming the effectiveness and safety of these drugs in periodontal surgical procedures are warranted.

## Introduction

Periodontal surgical procedures, including scaling and root planning, can cause prolonged intense pain (Pihlstrom et al., 1999; Rathore et al., 2024). Thus, patients undergoing periodontal surgeries can be managed using pharmacological strategies which promote greater comfort (Steffens et al., 2010; Giorgetti et al., 2018).

Preemptive analgesia has been employed for reducing and controlling pain and discomfort in patients postoperatively after periodontal invasive surgical procedures (Konuganti et al., 2015; Malamed, 2023). The term “preemptive” refers to a form of analgesia administered before the onset of pain stimuli to prevent or reduce subsequent pain (Garcia et al., 2021; Myers and Jeske, 2023).

Some studies suggest that pre-operative administration of different anti-inflammatory agents, such as steroidal or non-steroidal anti-inflammatory drugs (NSAIDs), can reduce the intensity of post-operative pain and the need for supplementary analgesics in more invasive oral procedures (Vicentini et al., 2018; Bhutani et al., 2019).

There are concerns over the use of NSAIDs which, while inhibiting cyclooxygenases (COX-1 and COX-2) may cause adverse reactions such as gastric irritation, renal and cardiovascular adverse effects (Harirforoosh et al., 2013). Moreover, NSAIDs are also one of the main drugs that can cause hypersensitivity reactions (Blanca-Lopez et al., 2019). Considering these possible implications, a systematic review was conducted to examine the available scientific evidence regarding the possible adverse effects and safety of NSAIDs in patients who take NSAIDs for 10 days or less to relieve pain (as usually occurs in more invasive dental procedures). It was observed that most patients who take NSAIDs for a short period are not at increased risk of developing cardiovascular, gastric, renal or respiratory adverse effects when compared to patients who have not been exposed to these drugs (Aminoshariae et al., 2016).

Therefore, some investigations have explored the preemptive use of more specific NSAIDs for COX-2 inhibition in dental surgical procedures (Peres et al., 2012; Piecuch, 2012; Xie et al., 2020). Additionally, systematic reviews have assessed the effectiveness of the use of steroidal anti-inflammatory drugs for preemptive analgesia in procedures such as third molar removal (Herrera-Briones et al., 2013; Cetira Filho et al., 2020).

In this context, doubts remain regarding the optimal choice of anti-inflammatory agent, dose and interval for preemptive use of the medication in periodontal surgical procedures to ensure treatment which improves post-operative pain control and prevents patient discomfort, while not exposing them to increased risk of complications (Choi et al., 2021).

A previous systematic review assessed the effectiveness of oral use of corticosteroids to control pain and swelling of patients undergoing third molar extraction, periodontal procedures or implant surgeries (Wagner et al., 2022). Recently, a clinical practice guideline for management of acute dental pain was published (Carrasco-Labra, et al., 2024). However, these guidelines were largely based on studies in patients after third molar extraction. Another recent publication evaluated systematic reviews regarding the effectiveness and safety of the preemptive use of anti-inflammatory and analgesic drugs in the management of postoperative pain, edema, and trismus in oral surgery. It was also noted that third molar surgery was the most studied procedure and that more randomized clinical trials are still needed (Pimenta et al., 2024). Moreover, systematic reviews assessing the effectiveness of preemptive use of oral steroidal and non-steroidal anti-inflammatory drugs in patients undergoing periodontal surgical procedures are still scarce.

Therefore, the present study assessed the preemptive use of oral steroidal and non-steroidal anti-inflammatory drugs in controlling patient post-operative pain and discomfort after periodontal surgical procedures with the aim of aiding professionals in decision-making for optimal management in effective and safe use of this medication.

## Methods

### Protocol and registration

This systematic review was reported according to the Cochrane Handbook for Systematic Reviews of Interventions (Higgins and Green, 2020) and the Preferred Reporting Items for Systematic Reviews and Meta-Analyses (PRISMA) statement (Page et al., 2021). The study protocol was registered (CRD42022324766) on the PROSPERO platform (https://www.crd.york.ac.uk/prospero/display_record.php?RecordID=324766).

### Eligibility criteria

These criteria were described using the Population, Intervention, Comparison, Outcome and Study type (PICOS) framework.

#### Inclusion criteria

Participants: adults aged ≥18 years who required periodontal surgical interventions, such as subgingival scaling, clinical crown augmentation, and grafts;

Intervention: preemptive analgesia using oral corticosteroids or NSAIDS;

Control: placebo or active control (other steroidal or nonsteroidal anti-inflammatory drug);

Outcomes: effectiveness and safety outcomes;

Study type: randomized controlled trials (RCT).

#### Exclusion criteria

Participants: pregnant or nursing women; patients in continuous use of anti-inflammatories or analgesics for at least 14 days prior to the study, or with a history of allergy or intolerance to these drugs, or in use of drugs that may affect the perception of pain. Individuals with a history of alcoholism or substance abuse.

### Outcomes assessed

Clinical trials must report at least one of the following primary outcomes: pain reduction or control and patient discomfort (for at least 8 h after the surgical procedures), swelling/edema, and trismus. Pain must be assessed using a visual analogue scale or other scale; and edema should be assessed by angles or distances between different facial landmarks; and trismus by interincisal distance.

Secondary outcomes evaluated were the occurrence of adverse drug reactions, need for clinical reintervention, and satisfaction with the treatment.

### Search method for identifying studies

#### Electronic search of databases

The following databases were searched: Cochrane Central Register of Controlled Trials (CENTRAL), MEDLINE (via PubMed), Web of Science, Excerpta Medica dataBASE (EMBASE), Biblioteca Virtual em Saúde (BVS) and the thesis database of the Coordenação de Aperfeiçoamento de Pessoal de Nível Superior - CAPES (Brazilian Catalogue of Theses and Dissertations – https://catalogodeteses.capes.gov.br/catalogo-teses/#!/).

The search by RCT registry was performed on ClinicalTrials.gov (www.clinicaltrials.gov) and ISRCTN Register (www.isrctn.com). There were no restrictions for language or publication date and a search of all studies published up until July 2023 was carried out.

#### Other reference search sources

Grey Literature Report (https://www.greylit.org/library/search) and OpenGrey (http://www.opengrey.eu/) were the grey literature sources searched.

A manual search was performed by searching the list of eligible studies, literature review studies and systematic review studies. No lead authors of the studies needed to be contacted to obtain the full text or the necessary information for the data extraction.

### Search strategy

The search was conducted using Medical Subject Headings (MeSH) terms, with the strategy adapted for each database (see Supplementary Material SA).

After executing the search strategies on each database, the researchers imported the results of each search into an EndNote^®^ library for removal of duplicates and selection of the studies for review.

### Study eligibility

Four reviewers (LPL, CBM, EGF, and TLM), working in pairs and independently, screened potentially relevant citations and abstracts and applied the selection criteria. Full texts of all potentially eligible articles were obtained. The same reviewers confirmed the eligibility of each article by reading the full text. Disagreements were resolved by consensus and, when necessary, a third reviewer was consulted for a final decision (RHLM).

### Data extraction

Pairs of reviewers (LPL, CBM, EGF and TLM), independently, performed the data extraction, using a Microsoft Excel form, standardized and pretexted for this step. Reviewers extracted patient data, methods, interventions and outcomes evaluated. Disagreements were resolved by consensus and, when necessary, arbitrated by a third reviewer (RHLM or LCL).

### Risk of bias

The version of the Cochrane Collaboration was used to assess risk of bias. The reviewers, in pairs and independently (LPL,CBM, EGF and TLM), assigned the risk of bias for each clinical trial according to the following criteria: sequence generation: was the allocation sequence adequately generated?; Allocation concealment: was allocation adequately concealed?; Blinding of participants and care providers for each main outcome: was the knowledge of the allocated treatment adequately prevented during the trial?; Blinding of outcome assessors for each main outcome: was the knowledge of the allocated treatment adequately prevented during the trial?; Incomplete outcome data for each main outcome: did more than 10% of participants withdraw, and were incomplete outcome data adequately addressed?; Selective outcome reporting: was there any suggestion of selective outcome reporting?; Other sources of bias: was the trial apparently free of other problems that could put it at high risk of bias?

The reviewers assigned response options of “definitely yes,” “probably yes,” “probably no,” and “definitely no” for each of the domains, with “definitely yes” and “probably yes” ultimately being assigned a low risk of bias, and “definitely no” and “probably no,” a high risk of bias (Akl et al., 2013). The reviewers settled disagreements by consensus and a third reviewer (RHLM or LCL) was consulted when necessary.

### Data synthesis and quality of evidence analysis

The results were summarized through narrative synthesis, since it was not possible to perform meta-analyses due to disparities among the clinical procedures, drugs and doses used. Further information regarding the methods adopted is described in the protocol registered as already mentioned.

## Results

### Search strategy results

**FIGURE 1:**
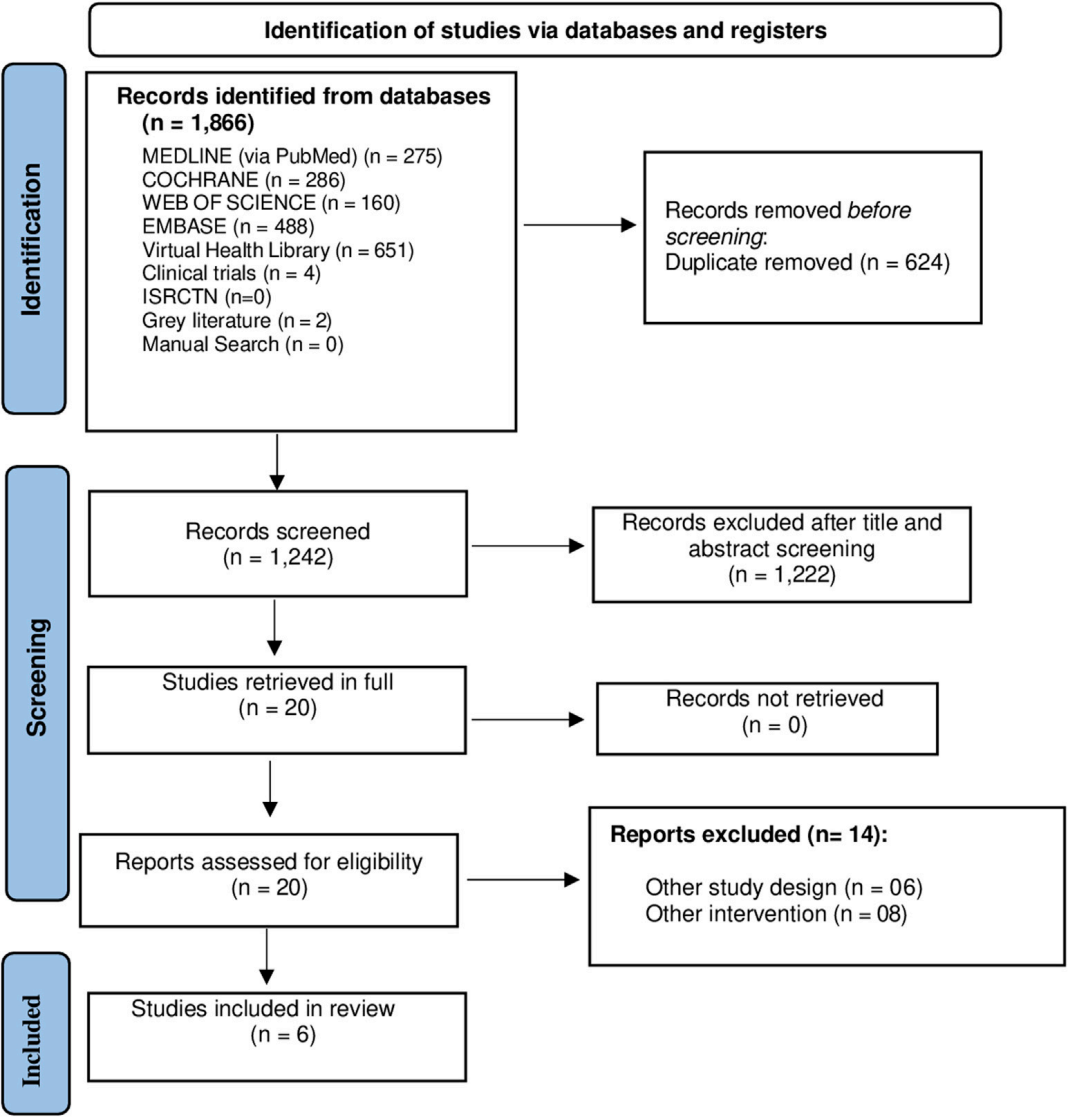
Flow diagram of study search process.

The search strategy led to the identification of 1,866 publications. After removal of duplicates and reading of titles and abstracts, 20 studies remained for full-text screening. Based on the eligibility criteria, a total of 6 RCTs were included in the review (Figure 1). The excluded studies are listed in Supplementary Material SB.

### Description of studies included

Six RCTs included involved 250 participants undergoing periodontal surgeries. Of the individuals for whom gender was reported, 75 were women and 57 men. Gender was not reported for the remaining 118 participants. The anti-inflammatory agents assessed in the clinical trials included dexamethasone 8 mg, celecoxib 200 mg, etoricoxib 90 mg and 120 mg, and ketorolac 20 mg. Patient follow-up ranged from the first 8 h to 4 days.

Of the 250 participants, 134 underwent flap debridement, 43 mucoperiosteal flap surgery, 15 root debridement and osseous recontouring, and 58 mucogingival grafts.

The studies included were published between 2010 and 2015. Four of the RCTs were conducted in Brazil, and the other 2 in India and Italy, respectively. The studies reported no information on research funding and did not register their protocols (Table 1).

**TABLE 1:** Characteristics of studies included (*n* = 6 studies).

Variables	Studies (n)	Population (n)
Population	6	250
Men	4	57
Women	4	75
Not reported	2	118
Medications		
Celecoxib 200 mg	2	26
Ketorolac 20 mg	1	22
Dexamethasone 8 mg	3	54
Etoricoxib 120 mg	3	55
Etoricoxib 90 mg	2	25
Placebo	6	98
Follow-up time		
1 day	1	18
1–4 days	5	232
Type of periodontal surgery		
Root debridement and osseous recontouring	1	15
Flap debridement	3	134
Gingival graft	1	58
Mucoperiosteal flap	1	43
Country		
Brazil	4	147
Italy	1	43
India	1	60
Year of publication		
1996–2010	2	58
2011–2015	4	192
Funding		
Not reported	6	250

### Risk of bias

**FIGURE 2:**
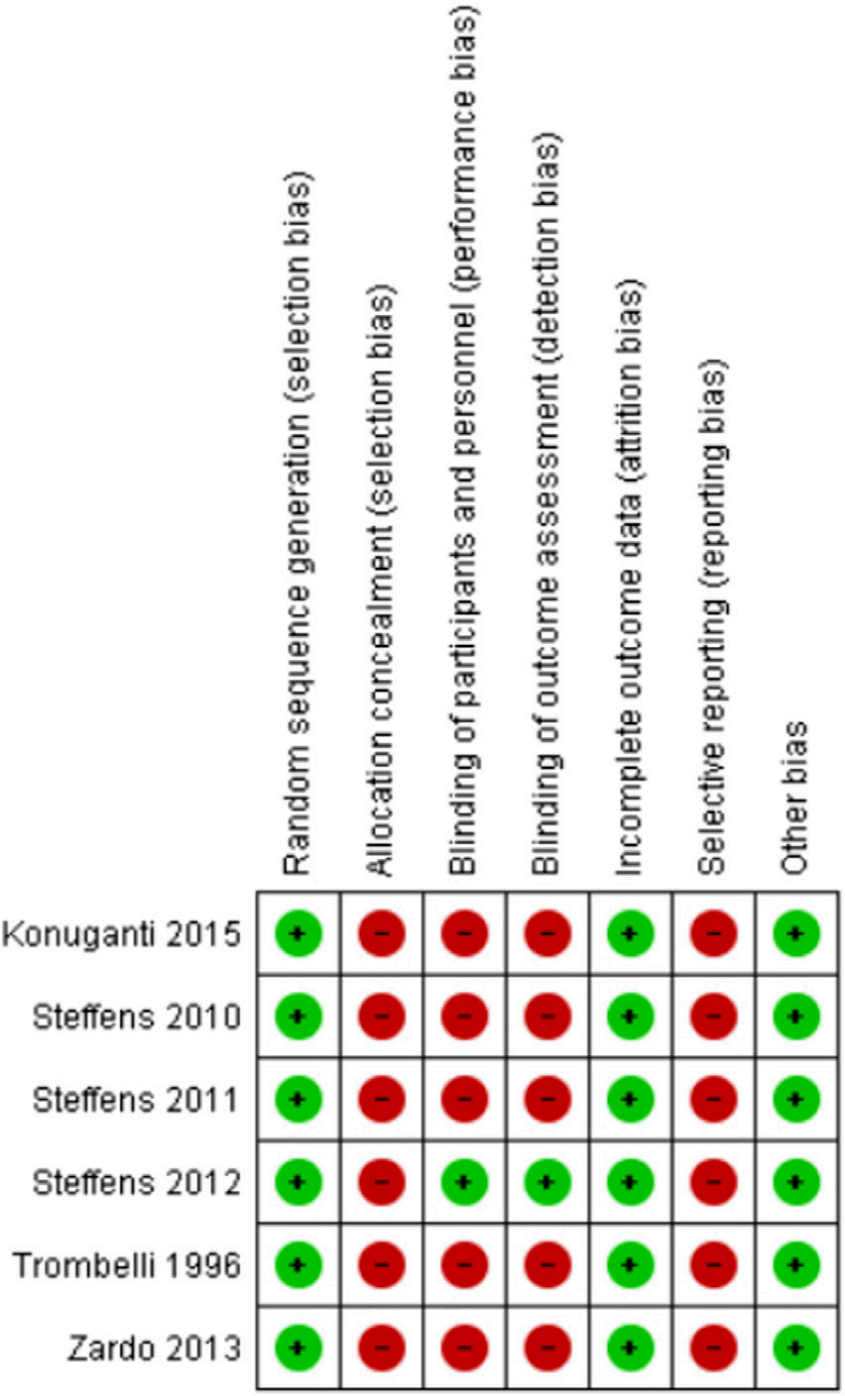
Risk of bias due to randomized clinical trial included.

Information about the risk of bias is described in Figure 2.

#### Random sequence generation

Two studies reported the methods used as random assigning by draw (Steffens et al., 2012) and randomization list (Trombelli et al., 1996). The other four studies stated that patients were randomized into groups but failed to describe the method used (Steffens et al., 2010; Steffens et al., 2011; Zardo et al., 2013; Konuganti et al., 2015).

#### Allocation concealment

None of the studies reported the approach used for concealment of allocation of participants and were therefore rated as high risk of bias.

#### Blinding of participants and personnel

One study described that designated a researcher solely to administer the drugs, thereby ensuring blinding of both patients and researchers (Steffens et al., 2012). The other studies claimed to be double-blind but provided no details on blinding procedures (Trombelli et al., 1996; Steffens et al., 2010; Steffens et al., 2011; Zardo et al., 2013; Konuganti et al., 2015).

#### Blinding of outcome assessors

One study reported details on blinding of outcome assessors (Steffens et al., 2012). The remaining studies reported no further information, indicating detection bias. (Trombelli et al., 1996; Steffens et al., 2010; Steffens et al., 2011; Zardo et al., 2013; Konuganti et al., 2015).

#### Incomplete outcome data

One of the studies reported loss of 5 (25%) of its total of 20 participants due to reasons including dentin hypersensitivity (*n* = 1) and moving to a new city (*n* = 4) (Steffens et al., 2010). In another RCT, 4 (6.6%) out of the total sample of 60 patients were lost for failing to attend post-operative return visits or not filling out the pain report form properly (Steffens et al., 2011). Another study reported loss of 5 (12%) out of the 43 participants because the pain questionnaire was not completed properly (Trombelli et al., 1996). Two individuals (3.3%) failed to return the pain score form in another study (Zardo et al., 2013).The remaining studies had no losses to follow-up (Steffens et al., 2012; Konuganti et al., 2015).

#### Selective reporting

None of the studies registered the protocol, precluding any check on whether all the proposed outcomes were measured.

#### Other sources of bias

All studies carried declarations stating there were no external sources of funding and, thus, were considered low risk of bias given the absence of potential conflict of interest regarding results.

### Outcome assessed

The studies reported the outcomes pain (*n* = 6) and anxiety (*n* = 2). The effectiveness of ketorolac 20 mg for reducing post-operative pain was compared to placebo (Trombelli et al., 1996) (Table 2). Three clinical trials assessed the use of dexamethasone 8 mg compared to etoricoxib (90 and 120 mg) and placebo for controlling post-operative pain (Steffens et al., 2010; Zardo et al., 2013; Konuganti et al., 2015). The use of celecoxib 200 mg was compared to etoricoxib (90 and 120 mg) and placebo for post-operative pain control (Steffens et al., 2011; Steffens et al., 2012). The effectiveness of ketorolac 20 mg for reducing post-operative pain was compared to placebo (Trombelli et al., 1996).

**TABLE 2:** Description of information from studies included and outcomes assessed (*n* = 6 studies, *n* = 250 participants).

Authors and publication date	Study objectives	Interventions (n) vs. comparator (n)	Sample (n)	Main findings
Konuganti et al. (2015)	To evaluate the efficacy of using dexamethasone 8 mg and etoricoxib 120 mg for pain prevention after open-flap debridement surgery	Dexamethasone 8 mg Etoricoxib 120 mg Placebo Follow-up: hourly for the first 8 h and three times a day on the following 3 days	60	Etoricoxib and dexamethasone were considered effective for pain and discomfort prevention after open-flap debridement surgeries compared to placebo during follow-up. The intake of rescue medication was lower in the etoricoxib and dexamethasone groups compared to placebo
Steffens et al. (2010)	To evaluate the efficacy of using etoricoxib and dexamethasone for pain prevention after open-flap debridement surgery	Dexamethasone 8 mg Etoricoxib 120 mg Placebo Follow-up: every hour for the first 8 h and three times a day in the next 3 days	15	Dexamethasone or etoricoxib was superior to placebo in follow-up of 4–8 h. The intake of rescue medication was significantly lower in the etoricoxib and dexamethasone groups compared to placebo
Steffens et al. (2011)	To evaluate the clinical efficacy of two selective cyclooxygenase-2 inhibitors on pain prevention after periodontal open-flap debridement surgery	Celecoxib 200 mg Etoricoxib 120 mg Placebo Follow-up: every hour for the first 8 h and three times a day on the following day	56	Celecoxib was only superior to placebo in follow-up of 3 h. Etoricoxib was superior to placebo in the follow-up period of 2–7 h. The frequency of rescue medication intake was significantly lower in the etoricoxib group compared to the placebo and celecoxib groups
Steffens et al. (2012)	To evaluate the effectiveness of two COX-2 selective non-steroidal anti-inflammatory drugs on pain control after open flap debridement	Celecoxib 200 mg Etoricoxib 90 mg Placebo Follow-up: every hour for 8 h after surgery	18	Etoricoxib was superior to placebo in follow-up of 1 and 3 h; celecoxib was not superior to placebo at none of the times evaluated
Trombelli et al. (1996)	To evaluate the efficacy of preoperative ketorolac compared to placebo for periodontal postoperative pain	Ketorolac 20 mg Placebo Follow-up: Immediately after and every hour for the first 10 h, and four times a day for the next 2 days	43	Compared to the placebo, ketorolac notably diminished pain intensity and postponed the onset of postoperative pain for up to 4 h of follow-up. The administration of ketorolac resulted in a significant prolongation of the interval between presurgical drug administration and the requirement for postoperative analgesics
Zardo et al. (2013)	To compare the use of etoricoxib and dexamethasone for postoperative pain prevention and control after mucogingival surgery	Dexamethasone 8 mg Etoricoxib 90 mg Placebo Follow-up: every hour for the first 8 h and three times a day in the next 3 days	58	Etoricoxib or dexamethasone were superior to placebo for postoperative pain prevention and control after mucogingival surgery. The intake of rescue medication was significantly lower in the etoricoxib and dexamethasone groups compared to placebo

The main findings for effectiveness reported by the clinical trials are outlined below:

#### Dexamethasone or etoricoxib versus placebo

Two clinical trials compared the effectiveness of the preemptive use of dexamethasone 8 mg or etoricoxib 120 mg to placebo in patients undergoing periodontal flap debridement surgery. The 101-point numeric rate scale and the four point verbal rating scale were used to rate post-operative pain and discomfort. In both trials, dexamethasone 8 mg and etoricoxib 120 mg proved to be more effective than placebo for controlling pain (Steffens et al., 2010; Konuganti et al., 2015). In one study, each patient underwent three surgical procedures at intervals of 30 days with different formulations (Steffens et al., 2010).

Another clinical trial compared the use of dexamethasone 8 mg or etoricoxib 90 mg to placebo for controlling post-operative pain in patients undergoing mucogingival surgery. The NRS-101 scale was used to rate post-operative pain. Dexamethasone 8 mg and etoricoxib 90 mg proved to be superior to placebo for reducing post-operative pain, and both drugs administered preoperatively resulted in a lower intake of rescue medication (Zardo et al., 2013).

#### Celecoxib or etoricoxib versus placebo

Two clinical trials compared the use of celecoxib 200 mg, and etoricoxib 90 mg and 120 mg to placebo for controlling post-operative pain in patients undergoing open-flap debridement procedures.

Celecoxib 200 mg and etoricoxib 120 mg were superior to placebo for controlling post-operative pain, and rescue medication intake was significantly less frequent in the etoricoxib group. In another study, etoricoxib 90 mg were superior to placebo and celecoxib for this outcome. The VAS was used to measure pain outcome in both studies (Steffens et al., 2011; Steffens et al., 2012).

#### Ketorolac versus placebo

Ketorolac 20 mg was compared to placebo for controlling post-operative pain in patients undergoing periodontal flap debridement surgeries. The preoperative ketorolac administration was more effective than placebo for reducing initial pain intensity and delayed the onset of postoperative pain (Trombelli et al., 1996).

## Discussion

### Main findings and comparison against the literature

The present study reviewed the available evidence on the effectiveness and safety of the use of preemptive analgesia in patients undergoing periodontal surgical procedures: open-flap debridement (Steffens et al., 2011; Steffens et al., 2012; Konuganti et al., 2015), mucoperiosteal flap (Trombelli et al., 1996), root debridement with osseous recontouring (Steffens et al., 2010) and gingival graft (Zardo et al., 2013). None of the studies reviewed met all the assessment criteria for risk of bias, where the main issues found pertained to allocation concealment and blinding of individuals involved. The most investigated drugs were dexamethasone 8 mg and etoricoxib 90 mg and 120 mg. Meta-analyses were not performed owing to the disparities in clinical procedures, drugs and doses studied.

Preemptive analgesia with dexamethasone 8 mg were more effective than placebo for controlling post-operative pain (Steffens et al., 2010; Zardo et al., 2013; Konuganti et al., 2015). Likewise, etoricoxib 90 mg or 120 mg were also superior to placebo for controlling post-operative pain (Steffens et al., 2010; Steffens et al., 2011; Steffens et al., 2012; Zardo et al., 2013; Konuganti et al., 2015). Ketorolac 20 mg was more effective than placebo for pain control (Trombelli et al., 1996).

None of the studies assessed adverse effects, precluding any conclusion on the safety of the interventions studied. Furthermore, the outcomes swelling, trismus, need for clinical reintervention and treatment satisfaction were not addressed in the clinical trials, limiting out findings about this topic on the preemptive use of analgesia in periodontal surgeries. Since stress and dental anxiety can vary among patients and potentially impact pain perception, the State-Trait Anxiety Inventory and Corah’s Dental Anxiety Scale were applied in two studies (Steffens et al., 2010; Steffens et al., 2011). However, there was no statistical difference between the patients in different groups of both studies.

Although no information was collected on the safety of using the drugs investigated by the studies, when administered as a single oral dose, these medications are likely to be safe for use. In addition, lost follow-up reported by some of the studies reviewed were for reasons unrelated to safety of the medications.

Notably, a previous systematic review assessed the effectiveness of the use of oral anti-inflammatory corticosteroids in patients undergoing third molar extraction, periodontal or implant surgeries. This review included three of the clinical trials included in our study (Wagner et al., 2022).

### Study strengths and limitations

This review was methodologically robust, employing explicit eligibility criteria, risk of bias rating, a broad comprehensive search of databases, and independent dual review of each study included.

The primary studies included were a factor limiting the review findings, given the methodological quality of the clinical trials, different comparators and doses and failure to address relevant clinical outcomes, ultimately preventing meta-analyses.

### Implications for clinical practice and research

This review provided a synthesis of the available evidence in the literature on the effectiveness of preemptive analgesia with oral use of dexamethasone 8 mg, etoricoxib (90 mg and 120 mg) and ketorolac 20 mg in periodontal surgical procedures. The findings suggest that preemptive oral use of these anti-inflammatory drugs appears to control post-operative pain and discomfort after periodontal surgical procedures. The study findings can help inform decision-making in dental practice regarding the control of post-operative pain induced by periodontal surgeries.

Considering the various limitations, further clinical trials involving more rigorous methodology and standardized methods of gathering outcome data should by conducted. These investigations could increase the reliability of findings, indicating which medications are deemed effective and safe for preemptive analgesia in periodontal surgical procedures.

## Conclusion

Preemptive analgesia with a single oral dose of dexamethasone 8 mg, etoricoxib (90 mg and 120) mg and ketorolac 20 mg appear to control post-operative pain in periodontal surgeries compared to the use of placebo. However, the evidence is insufficient to support the effectiveness and safety of these anti-inflammatory drugs for use in patients undergoing periodontal surgeries. In view of the limitations of this review such as small number of studies and participants, high risk of bias, different comparators across studies, lack of findings on the safety outcomes; further clinical trials should be carried out to confirm the effectiveness and safety of the use of anti-inflammatory medications in periodontal surgeries.

## Data Availability

The original contributions presented in the study are included in the article/Supplementary Material, further inquiries can be directed to the corresponding author.
