# Supplementary material for: Preemptive oral analgesia with steroidal and nonsteroidal anti-inflammatory drugs in periodontal surgery: a systematic review
Source: Front Pharmacol. 2024 Jul 15;15:1385401. doi: 10.3389/fphar.2024.1385401 (PMC11285104; doi:10.3389/fphar.2024.1385401)
Supplement: Supplementary file 1 [file DataSheet1.docx]

**Supplementary Material A - Search strategies in databases**

**MEDLINE (via PubMed) (n= 275)**

| **#1** | (Periodontal Debridement) OR (Periodontal Pocket Debridement) OR (Periodontal Pocket Debridements) OR (Periodontal Debridements) OR (Subgingival Curettage) OR (Subgingival Curettages) OR (Periodontal Epithelial Debridement) OR (Periodontal Epithelial Debridements) OR (Gingivectomy) OR (Gingivoplasty) OR (Widman flap) OR OR (Lip repositioning) OR (Crown lengthening) OR (Aesthetic crown lengthening) OR (Advanced flap) OR (Periodontal Surgery) OR (Surgical Flaps) OR (Gingival Hemorrhage/surgery) OR (Chronic Periodontitis/surgery) OR (gingival graft) |
| --- | --- |
| **#2** | (Adrenal Cortex Hormones) OR (Adrenal Cortex Hormone) OR Corticosteroids OR Corticosteroid OR Corticoids OR Corticoid OR Glucocorticoids OR Glucocorticoid OR (Glucocorticoid Effect) OR (Glucocorticoid Effects) OR Methylfluorprednisolone OR Dexamethasone OR Betamethasone OR Flubenisolone OR (Methylprednisolone Acetate) OR Methylprednisolone-21-acetate OR (Methylprednisolone 21 acetate) OR Acetyl-Methylprednisolone OR (Acetyl Methylprednisolone) OR Methylprednisolone OR 6-Methylprednisolone OR (6 Methylprednisolone) OR (Anti-inflammatory Agents) OR (Anti Inflammatory Agents) OR (Antiinflammatory Agent) OR (Antiinflammatory Agents) OR Anti-Inflammatories OR (Anti Inflammatories) OR Antiinflammatories OR (Anti-Inflammatory Agent) OR (Anti Inflammatory Agent) OR NSAID OR (Nonsteroidal Anti-Inflammatory Agent) OR (Nonsteroidal Anti Inflammatory Agent) OR NSAIDs OR (Nonsteroidal Antiinflammatory Agents) OR (Non-Steroidal Anti-Inflammatory Agents) OR (Non Steroidal Anti Inflammatory Agents) OR (Nonsteroidal Anti-Inflammatory Agents) OR (Nonsteroidal Anti Inflammatory Agents) OR (Non-Steroidal Anti- Inflammatory Agent) OR (Non Steroidal Anti Inflammatory Agent) OR (Anti- Inflammatory Analgesics) OR (Aspirin-Like Agents) OR (Aspirin Like Agents) OR (Aspirin-Like Agent) OR (Aspirin Like Agent) OR Ketorolac OR (Ketorolac Tromethamine) OR Piroxicam OR Nimesulide OR Ibuprofen OR Acetaminophen OR (Cyclooxygenase 2 Inhibitors) OR (Analgesics Non-Narcotic) OR Celecoxib OR Etoricoxibe |
| **#3** | Pain OR (Acute Pain) OR (Acute Pains) OR (Post-surgical Pain) OR (Post Surgical Pain) OR (Post-operative Pain) OR (Post operative Pain) OR (Postsurgical Pain) OR (Post-operative Pains) OR (Postoperative Pain) OR (Chronic Postoperative Pain) OR (Chronic Postoperative Pain) OR (Chronic Post-surgical Pain) OR (Chronic Post surgical Pain) OR (Chro Pain OR (Acute Pain) OR (Acute Pains) OR (Post-surgical Pain) OR (Post Surgical Pain) OR (Post-operative Pain) OR (Post operative Pain) OR (Postsurgical Pain) OR (Post-operative Pains) OR (Postoperative Pain) OR (Chronic Postoperative Pain) OR (Chronic Postoperative Pain) OR (Chronic Post-surgical Pain) OR (Chronic Post surgical Pain) OR (Chronic Postsurgical Pain) OR (Chronic Postsurgical Pains) OR (Persistent Postsurgical Pain) OR (Chronic Post-operative Pain) OR (Chronic Post operative Pain) OR (Acute Postoperative Pain) OR (Acute Post Operative Pain) OR (Acute Post-operative Pain) OR (Preoperative Care) OR (Preoperative Procedure) OR (Preoperative Procedures) OR (Preoperative Period) OR (Pre-Emptive Analgesia) OR Analgesia OR Preemptive OR Pre-emptive OR (Pre emptive) OR (preanesthetic medication) OR (Prevention) OR (Operative pain) OR (Preanesthetic Medication) OR (Postoperative/prevention) OR (Therapeutic use)nic Postsurgical Pain) OR (Chronic Postsurgical Pains) OR (Persistent Postsurgical Pain) OR (Chronic Post-operative Pain) OR (Chronic Post operative Pain) OR (Acute Postoperative Pain) OR (Acute Post Operative Pain) OR (Acute Post-operative Pain) OR (Preoperative Care) OR (Preoperative Procedure) OR (Preoperative Procedures) OR (Preoperative Period) OR (Pre-Emptive Analgesia) OR Analgesia OR Preemptive OR Pre-emptive OR (Pre emptive) OR (preanesthetic medication) OR (Prevention) OR (Operative pain) OR (Preanesthetic Medication) OR (Postoperative/prevention) OR (Therapeutic use) |
| **#4** | (randomized controlled trial[pt] OR controlled clinical trial[pt] OR randomized[tiab] OR placebo[tiab] OR clinical trials as topic[mesh:noexp] OR randomly[tiab] OR trial[tiab]) NOT (animals[mh] NOT humans [mh]) |
| **#5** | #1 AND #2 AND #3 AND #4 |

**Cochrane Library (n= 286)**

| **#1** | (Periodontal Debridement) OR (Periodontal Pocket Debridement) OR (Periodontal Pocket Debridements) OR (Periodontal Debridements) OR (Subgingival Curettage) OR (Subgingival Curettages) OR (Periodontal Epithelial Debridement) OR (Periodontal Epithelial Debridements) OR (Gingivectomy) OR (Gingivoplasty) OR (Widman flap) OR (Modified widman flap) OR (Lip repositioning) OR (Crown lengthening) OR (Aesthetic crown lengthening) OR (Advanced flap) OR (Periodontal Surgery) OR (Surgical Flaps) OR (gingival graft) |
| --- | --- |
| **#2** | (Adrenal Cortex Hormones) OR (Adrenal Cortex Hormone) OR Corticosteroids OR Corticosteroid OR Corticoids OR Corticoid OR Glucocorticoids OR Glucocorticoid OR (Glucocorticoid Effect) OR (Glucocorticoid Effects) OR Methylfluorprednisolone OR Dexamethasone OR Betamethasone OR Flubenisolone OR (Methylprednisolone Acetate) OR (Methylprednisolone 21 acetate) OR (Acetyl Methylprednisolone) OR Methylprednisolone OR (6 Methylprednisolone) OR (Anti-inflammatory Agents) OR (Anti Inflammatory Agents) OR (Antiinflammatory Agent) OR (Antiinflammatory Agents) OR Anti-Inflammatories OR (Anti Inflammatories) OR Antiinflammatories OR (Anti-Inflammatory Agent) OR (Anti Inflammatory Agent) OR NSAID OR (Nonsteroidal Anti-Inflammatory Agent) OR (Nonsteroidal Anti Inflammatory Agent) OR NSAIDs OR (Nonsteroidal Antiinflammatory Agents) OR (Non-Steroidal Anti-Inflammatory Agents) OR (Non Steroidal Anti Inflammatory Agents) OR (Nonsteroidal Anti-Inflammatory Agents) OR (Nonsteroidal Anti Inflammatory Agents) OR (Non-Steroidal Anti- Inflammatory Agent) OR (Non Steroidal Anti Inflammatory Agent) OR (Anti- Inflammatory Analgesics) OR (Aspirin-Like Agents) OR (Aspirin Like Agents) OR (Aspirin Like Agent) OR Ketorolac OR (Ketorolac Tromethamine) OR Piroxicam OR Nimesulide OR Ibuprofen OR Acetaminophen OR (Cyclooxygenase 2 Inhibitors) OR (Analgesics Non-Narcotic) OR Celecoxib OR Etoricoxibe |
| **#3** | Pain OR (Acute Pain) OR (Acute Pains) OR (Post-surgical Pain) OR (Post Surgical Pain) OR (Post-operative Pain) OR (Post operative Pain) OR (Postsurgical Pain) OR (Post-operative Pains) OR (Postoperative Pain) OR (Chronic Postoperative Pain) OR (Chronic Postoperative Pain) OR (Chronic Post-surgical Pain) OR (Chronic Post surgical Pain) OR (Chronic Postsurgical Pain) OR (Chronic Postsurgical Pains) OR (Persistent Postsurgical Pain) OR (Chronic Post-operative Pain) OR (Chronic Post operative Pain) OR (Acute Postoperative Pain) OR (Acute Post Operative Pain) OR (Acute Post-operative Pain) OR (Preoperative Care) OR (Preoperative Procedure) OR (Preoperative Procedures) OR (Preoperative Period) OR (Pre-Emptive Analgesia) OR Analgesia OR Preemptive OR Pre-emptive OR (Pre emptive) OR (preanesthetic medication) OR (Prevention) OR (Operative pain) OR (Preanesthetic Medication) OR (Therapeutic use) |
| **#4** | #1AND #2AND #3 |

**Web of Science (n= 160)**

| **#1** | TS= (Periodontal Debridement) OR (Periodontal Pocket Debridement) OR (Periodontal Pocket Debridements) OR (Periodontal Debridements) OR (Subgingival Curettage) OR (Subgingival Curettages) OR (Periodontal Epithelial Debridement) OR (Periodontal Epithelial Debridements) OR (Gingivectomy) OR (Gingivoplasty) OR (Widman flap) OR (Modified widman flap) OR (Lip repositioning) OR (Crown lengthening) OR (Aesthetic crown lengthening) OR (Advanced flap) OR (Periodontal Surgery) OR (Surgical Flaps) OR (Gingival Hemorrhage/surgery) OR (Chronic Periodontitis/surgery) OR (gingival graft) |
| --- | --- |
| **#2** | TS=((Adrenal Cortex Hormones) OR (Adrenal Cortex Hormone) OR Corticosteroids OR Corticosteroid OR Corticoids OR Corticoid OR Glucocorticoids OR Glucocorticoid OR (Glucocorticoid Effect) OR (Glucocorticoid Effects) OR Methylfluorprednisolone OR Dexamethasone OR Betamethasone OR Flubenisolone OR (Methylprednisolone Acetate) OR Methylprednisolone-21-acetate OR (Methylprednisolone 21 acetate) OR Acetyl-Methylprednisolone OR (Acetyl Methylprednisolone) OR Methylprednisolone OR 6-Methylprednisolone OR (6 Methylprednisolone) OR (Anti-inflammatory Agents) OR (Anti Inflammatory Agents) OR (Antiinflammatory Agent) OR (Antiinflammatory Agents) OR Anti-Inflammatories OR (Anti Inflammatories) OR Antiinflammatories OR (Anti-Inflammatory Agent) OR (Anti Inflammatory Agent) OR NSAID OR (Nonsteroidal Anti-Inflammatory Agent) OR (Nonsteroidal Anti Inflammatory Agent) OR NSAIDs OR (Nonsteroidal Antiinflammatory Agents) OR (Non-Steroidal Anti-Inflammatory Agents) OR (Non Steroidal Anti Inflammatory Agents) OR (Nonsteroidal Anti-Inflammatory Agents) OR (Nonsteroidal Anti Inflammatory Agents) OR (Non-Steroidal Anti-Inflammatory Agent) OR (Non Steroidal Anti Inflammatory Agent) OR (Anti-Inflammatory Analgesics) OR (Aspirin-Like Agents) OR (Aspirin Like Agents) OR (Aspirin-Like Agent) OR (Aspirin Like Agent) OR Ketorolac OR (Ketorolac Tromethamine) OR Piroxicam OR Nimesulide OR Ibuprofen OR Acetaminophen OR (Cyclooxygenase 2 Inhibitors) OR (Analgesics Non-Narcotic) OR Celecoxib OR Etoricoxib) |
| **#3** | TS=(Pain OR (Acute Pain) OR (Acute Pains) OR (Post-surgical Pain) OR (Post Surgical Pain) OR (Post-operative Pain) OR (Post operative Pain) OR (Postsurgical Pain) OR (Post-operative Pains) OR (Postoperative Pain) OR (Chronic Postoperative Pain) OR (Chronic Postoperative Pain) OR (Chronic Post-surgical Pain) OR (Chronic Post surgical Pain) OR (Chronic Postsurgical Pain) OR (Chronic Postsurgical Pains) OR (Persistent Postsurgical Pain) OR (Chronic Post-operative Pain) OR (Chronic Post operative Pain) OR (Acute Postoperative Pain) OR (Acute Post Operative Pain) OR (Acute Post-operative Pain) OR (Preoperative Care) OR (Preoperative Procedure) OR (Preoperative Procedures) OR (Preoperative Period) OR (Pre-Emptive Analgesia) OR Analgesia OR Preemptive OR Pre-emptive OR (Pre emptive) OR (preanesthetic medication) OR (Prevention) OR (Operative pain) OR (Preanesthetic Medication) OR (Postoperative/prevention) OR (Therapeutic use)) |
| **#4** | **#1 AND #2 AND #3** |

**EMBASE (n= 488)**

| **#1** | (periodontal AND debridement OR (periodontal AND pocket AND debridement) OR (periodontal AND pocket AND debridements) OR (periodontal AND debridements) OR (subgingival AND curettage) OR (subgingival AND curettages) OR (periodontal AND epithelial AND debridement) OR (periodontal AND epithelial AND debridements) OR gingivectomy OR gingivoplasty OR (widman AND flap) OR (modified AND widman AND flap) OR (lip AND repositioning) OR (crown AND lengthening) OR (aesthetic AND crown AND lengthening) OR (advanced AND flap) OR (periodontal AND surgery) OR (surgical AND flaps) OR (gingival AND graft)) AND (adrenal AND cortex AND hormones OR (adrenal AND cortex AND hormone) OR corticosteroids OR corticosteroid OR corticoids OR corticoid OR glucocorticoids OR glucocorticoid OR (glucocorticoid AND effect) OR (glucocorticoid AND effects) OR methylfluorprednisolone OR dexamethasone OR betamethasone OR flubenisolone |
| --- | --- |
| **#2** | (Adrenal Cortex Hormones) OR (Adrenal Cortex Hormone) OR Corticosteroids OR Corticosteroid OR Corticoids OR Corticoid OR Glucocorticoids OR Glucocorticoid OR (Glucocorticoid Effect) OR (Glucocorticoid Effects) OR Methylfluorprednisolone OR Dexamethasone OR Betamethasone OR Flubenisolone OR (Methylprednisolone Acetate) OR (Methylprednisolone 21 acetate) OR (Acetyl Methylprednisolone) OR Methylprednisolone OR (6 Methylprednisolone) OR (Anti-inflammatory Agents) OR (Anti Inflammatory Agents) OR (Antiinflammatory Agent) OR (Antiinflammatory Agents) OR Anti-Inflammatories OR (Anti Inflammatories) OR Antiinflammatories OR (Anti-Inflammatory Agent) OR (Anti Inflammatory Agent) OR NSAID OR (Nonsteroidal Anti-Inflammatory Agent) OR (Nonsteroidal Anti Inflammatory Agent) OR NSAIDs OR (Nonsteroidal Antiinflammatory Agents) OR (Non-Steroidal Anti-Inflammatory Agents) OR (Non Steroidal Anti Inflammatory Agents) OR (Nonsteroidal Anti-Inflammatory Agents) OR (Nonsteroidal Anti Inflammatory Agents) OR (Non-Steroidal Anti-Inflammatory Agent) OR (Non Steroidal Anti Inflammatory Agent) OR (Anti-Inflammatory Analgesics) OR (Aspirin-Like Agents) OR (Aspirin Like Agents) OR (Aspirin Like Agent) OR Ketorolac OR (Ketorolac Tromethamine) OR Piroxicam OR Nimesulide OR Ibuprofen OR Acetaminophen OR (Cyclooxygenase 2 Inhibitors) OR (Analgesics Non-Narcotic) OR Celecoxib OR Etoricoxib |
| **#3** | Pain OR (Acute Pain) OR (Acute Pains) OR (Post-surgical Pain) OR (Post Surgical Pain) OR (Post-operative Pain) OR (Post operative Pain) OR (Postsurgical Pain) OR (Post-operative Pains) OR (Postoperative Pain) OR (Chronic Postoperative Pain) OR (Chronic Postoperative Pain) OR (Chronic Post-surgical Pain) OR (Chronic Post surgical Pain) OR (Chronic Postsurgical Pain) OR (Chronic Postsurgical Pains) OR (Persistent Postsurgical Pain) OR (Chronic Post-operative Pain) OR (Chronic Post operative Pain) OR (Acute Postoperative Pain) OR (Acute Post Operative Pain) OR (Acute Post-operative Pain) OR (Preoperative Care) OR (Preoperative Procedure) OR (Preoperative Procedures) OR (Preoperative Period) OR (Pre-Emptive Analgesia) OR Analgesia OR Preemptive OR Pre-emptive OR (Pre emptive) OR (preanesthetic medication) OR (Prevention) OR (Operative pain) OR (Preanesthetic Medication) OR (Therapeutic use) |
| **#4** | **#1 AND #2 AND #3** |

**Virtual Health Library (n= 651)**

| **#1** | (Periodontal Debridement) OR (Periodontal Pocket Debridement) OR (Periodontal Pocket Debridements) OR (Periodontal Debridements) OR (Subgingival Curettage) OR (Subgingival Curettages) OR (Periodontal Epithelial Debridement) OR (Periodontal Epithelial Debridements) OR (Gingivectomy) OR (Gingivoplasty) OR (Widman flap) OR (Modified widman flap) OR (Lip repositioning) OR (Crown lengthening) OR (Aesthetic crown lengthening) OR (Advanced flap) OR (Periodontal Surgery) OR (Surgical Flaps) OR (Gingival Hemorrhage/surgery) OR (Chronic Periodontitis/surgery) OR (gingival graft) |
| --- | --- |
| **#2** | (Adrenal Cortex Hormones) OR (Adrenal Cortex Hormone) OR Corticosteroids OR Corticosteroid OR Corticoids OR Corticoid OR Glucocorticoids OR Glucocorticoid OR (Glucocorticoid Effect) OR (Glucocorticoid Effects) OR Methylfluorprednisolone OR Dexamethasone OR Flubenisolone OR (Methylprednisolone Acetate) OR Methylprednisolone-21-acetate OR (Methylprednisolone 21 acetate) OR Acetyl-Methylprednisolone OR (Acetyl Methylprednisolone) OR Methylprednisolone OR 6-Methylprednisolone OR (6 Methylprednisolone) OR (Anti-inflammatory Agents) OR (Anti Inflammatory Agents) OR (Antiinflammatory Agent) OR (Antiinflammatory Agents) OR Anti-Inflammatories OR (Anti Inflammatories) OR Antiinflammatories OR (Anti-Inflammatory Agent) OR (Anti Inflammatory Agent) OR NSAID OR (Nonsteroidal Anti-Inflammatory Agent) OR (Nonsteroidal Anti Inflammatory Agent) OR NSAIDs OR (Nonsteroidal Antiinflammatory Agents) OR (Non-Steroidal Anti-Inflammatory Agents) OR (Non Steroidal Anti Inflammatory Agents) OR (Nonsteroidal Anti-Inflammatory Agents) OR (Nonsteroidal Anti Inflammatory Agents) OR (Non-Steroidal Anti-Inflammatory Agent) OR (Non Steroidal Anti Inflammatory Agent) OR (Anti-Inflammatory Analgesics) OR (Aspirin-Like Agents) OR (Aspirin Like Agents) OR (Aspirin-Like Agent) OR (Aspirin Like Agent) OR Ketorolac OR (Ketorolac Tromethamine) OR Piroxicam OR Nimesulide OR Ibuprofen OR Acetaminophen OR (Cyclooxygenase 2 Inhibitors) OR (Analgesics Non-Narcotic) OR Celecoxib OR Etoricoxib OR (Dexketoprofen Trometamol) OR (Sublingual Piroxicam) OR Meloxicam OR Celebra OR COX-2 OR (COX-2 Inhibitors) OR Analgesics OR (Anti-inflammatory Drugs) |
| **#3** | Pain OR (Acute Pain) OR (Acute Pains) OR (Post-surgical Pain) OR (Post Surgical Pain) OR (Post-operative Pain) OR (Post operative Pain) OR (Postsurgical Pain) OR (Post-operative Pains) OR (Postoperative Pain) OR (Chronic Postoperative Pain) OR (Chronic Postoperative Pain) OR (Chronic Post-surgical Pain) OR (Chronic Post surgical Pain) OR (Chronic Postsurgical Pain) OR (Chronic Postsurgical Pains) OR (Persistent Postsurgical Pain) OR (Chronic Post-operative Pain) OR (Chronic Post operative Pain) OR (Acute Postoperative Pain) OR (Acute Post operative Pain) OR (Acute Post-operative Pain) OR (Preoperative Care) OR (Preoperative Procedure) OR (Preoperative Procedures) OR (Preoperative Period) OR Preemptive OR Pre-emptive OR (Pre emptive) OR Analgesia OR (Pre-Emptive Analgesia) OR (Preanesthetic Medication) OR Prevention OR (Operation Pain) OR (Postoperative/prevention) OR (Therapeutic Use) OR (Preemptive Analgesia) OR (Postoperative/Drug Therapy) OR Edema OR Inflammation |
| **#4** | *(db:("LILACS") AND type_of_study:("randomized controlled trial"))* |

**CLINICAL TRIALS (n= 04)**

| **#1** | ((Periodontal Debridement) OR (Periodontal Pocket Debridement) OR (Subgingival Curettage) OR (Gingivectomy) OR (Gingivoplasty) OR (Widman flap) OR (Modified widman flap) OR (Lip repositioning) OR (Crown lengthening) OR (Periodontal Surgery)) |
| --- | --- |
| **#2** | ((Corticosteroids) OR (Corticoids) OR (Dexamethasone) OR (Betamethasone) OR (NSAID) OR (Nonsteroidal Anti-Inflammatory Agent) OR (NSAIDs) OR (Ketorolac) OR (Piroxicam) OR (Nimesulide) OR (Ibuprofen) OR (Acetaminophen) OR (Celecoxib)) |

**ISRCTN (n= 00)**

| **#1Doença** | ((Periodontal Debridement) OR (Periodontal Pocket Debridement) OR (Subgingival Curettage) OR (Gingivectomy) OR (Gingivoplasty) OR (Widman flap) OR (Modified widman flap) OR (Lip repositioning) OR (Crown lengthening) OR (Periodontal Surgery)) |
| --- | --- |
| **#2Intervenção** | ((Corticosteroids) OR (Corticoids) OR (Dexamethasone) OR (Betamethasone) OR (NSAID) OR (Nonsteroidal Anti-Inflammatory Agent) OR (NSAIDs) OR (Ketorolac) OR (Piroxicam) OR (Nimesulide) OR (Ibuprofen) OR (Acetaminophen) OR (Celecoxib)) |

**GREY LITERATURE (n= 02)**

| **The New York Academy of Medicine Library Online Catalog** | [**https://catalog.nyam.org/**](https://catalog.nyam.org/)  Preemptive Analgesia, Pre-emptive analgesia, Preemptive, Pre-emptive, Analgesia, Periodontal Debridement, Periodontal Pocket Debridement, Subgingival Curettage, Gingivectomy, Gingivoplasty, Widman flap, Modified widman flap, Lip repositioning, Crown lengthening, Periodontal Surgery |
| --- | --- |
| **Catalog of Theses and Dissertations of Brazil (n= 02)** | [**https://catalogodeteses.capes.gov.br/catalogo-teses/#!/**](https://catalogodeteses.capes.gov.br/catalogo-teses/#!/)  Analgesia preemptiva (área de conhecimento – ODONTOLOGIA) |

**Supplementary Material B – LIST OF EXCLUDED STUDIES (N= 14)**

| **References** | **Reason for exclusion** |
| --- | --- |
| Anil A, Gujjari SK, Venkatesh MP. Evaluation of a curcumin-containing mucoadhesive film for periodontal postsurgical pain control. J Indian  Soc Periodontol. 2019 Sep-Oct;23(5):461-468. | The intervention was not preemptive |
| Bateman GJ, Saha S, Pearson D. Contemporary periodontal surgery:  2. Surgical practice. Dent Update. 2008 Sep;35(7):470-2, 475-6, 478. | Another type of study |
| Betancourt JW, Kupp LI, Jasper SJ, Farooqi OA. Efficacy of ibuprofen- hydrocodone for the treatment of postoperative pain after periodontal  surgery. J Periodontol. 2004 Jun;75(6):872-6. | The intervention was not preemptive |
| Braganza A, Bissada N, Hatch C, Ficara A. The effect of non-steroidal anti-inflammatory drugs on bleeding during periodontal surgery. J  Periodontol. 2005 Jul;76(7):1154-60. | Another control group |
| Bragger U, Muhle T, Fourmousis I, Mombelli A. Effect of the NSAID  flurbiprofen on remodelling after periodontal surgery. J Periodont Res 1997; 32:575-582. | The intervention was not preemptive |
| Burgos-Quirîz, K. M.; Asmat-Abanto, A. S.; Espejo-Carrera, R. E.  Efectividad analgesica de paracetamol  post-cirugía mucogingival: ensayo clínico aleatorizado paralelo. Int. J. Odontostomat., 13(2):184-188, 2019. | The intervention was not preemptive |
| Cooper SA, Wagenberg B, Eskow R, Zissu J. Double-blind evaluation of suprofen and aspirin in the treatment of periodontal pain.  Pharmacology. 1983;27 Suppl 1:23-30. | The intervention was not preemptive |
| Cooper SA. Five studies on ibuprofen for postsurgical dental pain. Am J Med. 1984 Jul 13;77(1A):70-7. | Another type of study |
| Cooper SA, Wagenberg B, Zissu J, Kruger GO, Reynolds DC, Gallegos LT, Allwein JB, Desjardins PJ, Friedmann N, Danna RP. The analgesic efficacy of suprofen in periodontal and oral surgical pain.  Pharmacotherapy. 1986 Sep-Oct;6(5):267-76. | The intervention was not preemptive |
| Das R, Deshmukh J, Asif K, Sindhura H, Devarathanamma MV, Jyothi  L. Comparative evaluation of analgesic and anti-inflammatory efficacy of ibuprofen and traumeel after periodontal flap surgery: A randomized  triple-blind clinical trial. J Indian Soc Periodontol. 2019 Nov- Dec;23(6):549-553. | The intervention was not preemptive |
| Dionne RA, Berthold CW. Therapeutic uses of non-steroidal anti- inflammatory drugs in dentistry. Crit Rev Oral Biol Med.  2001;12(4):315-30. | Another type of study |
| Diwan V, Srinivasa TS, Ramreddy KY, Agrawal V, Nagdeve S, Parvez  H. A comparative evaluation of transdermal diclofenac patch with oral diclofenac sodium as an analgesic drug following periodontal flap surgery: A randomized controlled clinical study. Indian J Dent Res.  2019 Jan-Feb;30(1):57-60. | The intervention was not preemptive |
| Etikala A, Tattan M, Askar H, Wang HL. Effects of NSAIDs on  Periodontal and Dental Implant Therapy. Compend Contin Educ Dent. 2019 Feb;40(2):e1-e9. | Another type of study |
| Fang M, He J, Ma X, Li W, Lin D. Protective effects of  dexmedetomidine on the survival of random flaps. Biomed Pharmacother. 2020 Aug;128:110261 | Another type of study |
